# Efficacy and safety of daratumumab in the treatment of relapsed/refractory multiple myeloma: A meta-analysis of randomized controlled trials

**DOI:** 10.1097/MD.0000000000035319

**Published:** 2023-09-22

**Authors:** Zeng-Yi Huang, Xiao-Qin Jin, Qi-Lian Liang, Ding-Yue Zhang, Han Han, Zhen-Wei Wang

**Affiliations:** a Oncology Center, Affiliated Hospital of Guangdong Medical University, Zhanjiang, China.

**Keywords:** daratumumab, meta-analysis, relapsed or refractory multiple myeloma

## Abstract

**Background:**

Daratumumab as a monoclonal antibody has shown promising results in the treatment of relapsed/refractory multiple myeloma (RRMM). However, the efficacy and safety of daratumumab-based regimens compared to control regimens have not been fully established.

**Methods:**

The search was conducted using electronic databases (PubMed, Web of Science, Embase, and Cochrane Central Register of Controlled Trials databases) up to December 2022. We conducted a meta-analysis of randomized controlled trials that evaluated the efficacy and safety of daratumumab in the treatment of RRMM. Data were extracted from eligible studies and were presented as hazard ratio or risk ratio (RR) with 95% confidence interval (CI).

**Results:**

A total of 5 randomized controlled trials comprising 2003 patients were included in this meta-analysis. The results showed that daratumumab-based regimens significantly improved progression-free survival compared to control regimens (hazard ratio = 0.44, 95% CI 0.32–0.60, *P* < .00001). Additionally, daratumumab-based regimens significantly improved overall response rate compared to control regimens (RR = 1.25, 95% CI 1.16–1.36, *P* < .00001). the rate of minimal residual disease was also significantly higher in the daratumumab-based regimens (RR = 6.10, 95% CI 4.09–9.11, *P* < .00001). However, there was an increased risk of pneumonia, upper respiratory tract infections, and diarrhea in the daratumumab-based regimens.

**Conclusion:**

Our results suggest that daratumumab-based regimens are effective in the treatment of RRMM, improving progression-free survival, minimal residual disease, and overall response rate. However, there is an increased risk of pneumonia, upper respiratory tract infections, and diarrhea. Further studies are needed to determine the long-term safety and efficacy of daratumumab in the treatment of multiple myeloma.

## 1. Introduction

Multiple myeloma (MM) is a malignancy characterized by the abnormal proliferation of clonal plasma cells. MM accounts for approximately 1% of all cancers and 10% to 15% of all hematological tumors worldwide.^[1–3]^ The incidence of MM, the second-most common hematologic malignancy in adults, is primarily observed in elderly individuals, with a median age at diagnosis of 69 years.^[4–6]^ The disease is characterized by the proliferation of abnormal plasma cells, which accumulate in the bone marrow and disrupt normal hematopoiesis.^[7–9]^ MM is associated with significant morbidity and mortality, and it poses a substantial burden on patients and healthcare systems worldwide.^[10,11]^

Despite advances in the understanding and treatment of MM, the disease remains incurable, and patients often experience relapse or disease progression after initial therapy.^[12–15]^ refractory or relapsed multiple myeloma (RRMM) is a particularly difficult clinical scenario, as patients have limited treatment options and poor prognosis.^[16]^

The development of new therapies that target the underlying biology of MM is critical for improving outcomes in RRMM. CD38 is a transmembrane glycoprotein that is highly expressed on the surface of malignant plasma cells in MM patients.^[17,18]^ It plays a critical role in the regulation of cell signaling, adhesion, and immune response, and its expression level has been identified as a potential therapeutic target in MM.^[17,19,20]^ Daratumumab is a human monoclonal antibody that targets CD38 on the surface of malignant plasma cells in MM patients.^[21,22]^ It has demonstrated significant clinical activity in patients with relapsed or refractory disease, leading to the destruction of tumor cells through multiple mechanisms.^[19,23]^

Despite the promising results of daratumumab in clinical trials, its application for patients with RRMM has been limited. There is a need for further research to evaluate the efficacy and safety of daratumumab in this patient population and to explore its potential role in combination with other agents.

In this study, we aim to evaluate the clinical outcomes of daratumumab in patients with RRMM and provide a theoretical foundation for the treatment of these patients. We believe that our findings will contribute to the development of new therapeutic strategies for MM and improve outcomes for patients with this challenging disease.

## 2. Materials and methods

This systematic review and meta-analysis were conducted in accordance with the Preferred Reporting Items for Systematic Reviews and Meta-Analyses guidelines. This meta-analysis was registered at PROSPERO, #CRD42022371406.

### 2.1. Search strategy

We conducted a comprehensive search of PubMed, Web of Science, Embase, and the Cochrane Central Register of Controlled Trials databeses, from their inception up to December 1, 2022. The search strategy included the following terms: daratumumab, multiple myeloma, relapsed/refractory, randomized controlled trials, and their relevant combinations. The search was limited to studies conducted in humans and published in English. In addition, we manually searched the reference lists of identified articles and relevant reviews to identify any additional studies that met our inclusion criteria. Two independent reviewers screened the articles for eligibility, and any disagreements were resolved through discussion or consultation with a third reviewer.

### 2.2. Inclusion criteria

1. Randomized controlled trials (RCTs) that evaluated the use of daratumumab in the treatment of RRMM.
2. Studies that reported the overall response rate (ORR), complete response (CR) rate, progression-free survival (PFS), or minimal residual disease (MRD) negativity rate as outcome measures.
3. Studies that compared daratumumab-containing regimens with non-daratumumab-containing regimens in the treatment of RRMM.
4. Studies that included adult patients (age ≥ 18 years) with confirmed diagnosis of RRMM.

### 2.3. Exclusion criteria

1. Non-randomized controlled trials. Studies that did not report the outcomes of interest, such as ORR, CR rate, PFS, or MRD negativity rate.
2. Studies that did not involve the use of daratumumab or did not compare daratumumab-containing regimens with non-daratumumab-containing regimens.
3. Studies that included pediatric patients or patients with other types of malignancies.

### 2.4. The following outcome measures were evaluated in this meta-analysis

1. ORR: The proportion of patients who achieved a partial response (PR) or better, according to the International Myeloma Working Group criteria.^[24]^
2. CR rate: The proportion of patients who achieved a CR, according to the International Myeloma Working Group criteria.^[24]^
3. PFS: The length of time from the start of treatment to disease progression or death.
4. MRD negativity rate: The proportion of patients who achieved MRD negativity after treatment, as determined by flow cytometry or polymerase chain reaction.^[24]^
5. Grade 3 to 4 hematologic toxicities: The incidence of Grade 3 to 4 neutropenia, lymphopenia, and thrombocytopenia, as defined by the common terminology criteria for adverse events.^[25]^

Non-hematologic toxicities: The incidence of pneumonia, upper respiratory tract infection, and diarrhea, as defined by the common terminology criteria for adverse events.^[25]^

### 2.5. Selection of studies and data extraction

This meta-analysis followed the guidelines provided by the Preferred Reporting Items for Systematic Reviews and Meta-Analyses report^[26]^ to ensure adherence to best practices. Data extraction was carried out independently by 2 authors, Ding–Yue Zhang and Zhen–Wei Wang. In the case of any disagreement, a third reviewer was available for consultation. The information that was collected included: Study characteristics, such as primary author, year of publication/time period of study, and study design; Characteristics of participants and RRMM, including the number of cases and participant age; and Interventions, comparators, reported results, methods of data pooling, estimation of effect size, heterogeneity, publication bias, and any other relevant details. When multiple studies contained the same or overlapping data, only the latest or higher quality studies were included to avoid duplication and ensure accuracy.

### 2.6. Assessment of risk of bias

The risk of bias for each of the included studies was assessed using the Cochrane Risk of Bias tool.^[27]^ The following domains were evaluated: random sequence generation, allocation concealment, blinding of participants, and personnel, blinding of outcome assessment, incomplete outcome data, selective reporting, and other biases. Two independent reviewers conducted the risk of bias assessment for each included study, and any disagreements were resolved through discussion and consensus. Each study was categorized as having a low, unclear, or high risk of bias for each domain.

The overall risk of bias for each study was evaluated as low, moderate, or high based on the assessment of all domains. Studies were considered to have a low risk of bias if all domains were rated as low risk, moderate risk of bias if 1 or more domains were rated as unclear or high risk, and high risk of bias if 1 or more domains were rated as high risk. The results of the risk of bias assessment were used to inform the interpretation of the meta-analysis results and the strength of the conclusions drawn from the pooled data.

### 2.7. Statistical analysis

For this meta-analysis, we used Review Manager 5.3 and Stata 14.0 software (https://www.stata.com/) to perform the statistical analysis. We calculated the risk ratio (RR) and 95% confidence intervals (CIs) to express dichotomous data and the hazard ratio (HR) and 95% CIs to express time-to-event data. We evaluated the heterogeneity among the results of the included trials using the Cochrane Q statistic and *I*^2^ value. If the *P* value was < .10 or the *I*^2^ value was > 50%, we considered the assumption of homogeneity to be invalid, and we used the Mantel–Haenszel random-effects model after exploring the causes of heterogeneity. Otherwise, we conducted a meta-analysis using a fixed-effect model. We considered a *P* value < .05 to be statistically significant for all outcomes. Additionally, we performed sensitivity analyses to assess the robustness of the results and publication bias was assessed using funnel plots and Egger test.

## 3. Results

### 3.1. Search results

**Figure 1.**
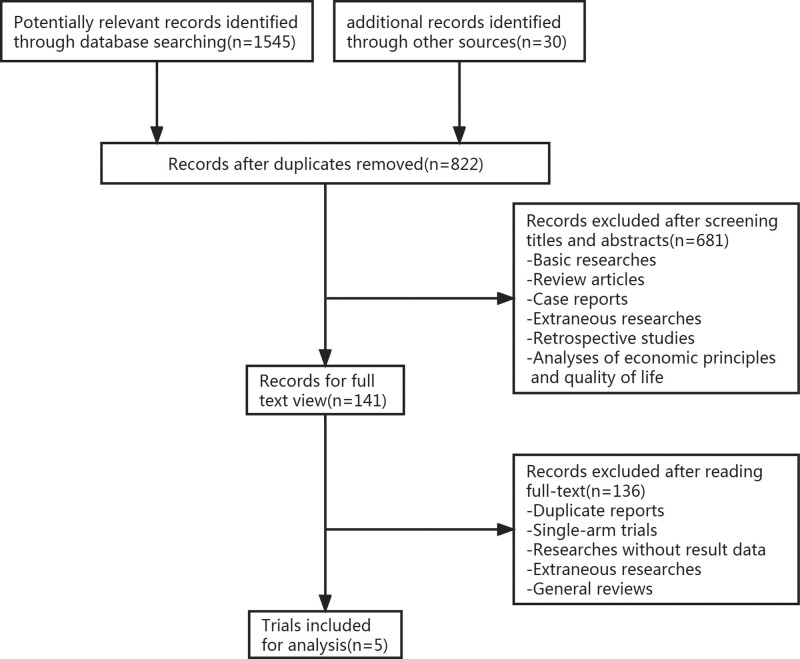
Flow diagram depicting the study selection process.

A total of 1575 potentially relevant records were identified in the initial search, of which 753 were duplicates. After screening titles and abstracts, 681 records were excluded due to not meeting the inclusion criteria, including basic research, review articles, case reports, retrospective studies, analyses of economic principles, and quality of life studies. Eventually, 5 randomized controlled trials were included in the meta-analysis.^[28–32]^ The search and selection process is presented in Figure 1.

### 3.2. Study characteristics and quality assessment

The meta-analysis included 5 RCTs with a total of 2003 patients who had relapsed or refractory multiple myeloma. The basic characteristics of the included studies were summarized in Table 1.

**Table 1:** Main characteristics of the five selected studies in this meta-analysis.

Study, year	Study design	Registration number	Number of patients	Prior lines of therapy	Regimens	Median age, years	Dose of daratumumab, mg
Bahlis et al 2020	RCT	NCT02076009	286:283	≥1	DRd vs Rd	65	16
Dimopoulos et al 2020	RCT	NCT03158688	312:154	≥1	KdD vs Kd	64/64.5	8/16
Dimopoulos et al 2021	RCT	NCT03180736	151:153	≥1	DPd vs Pd	67/68	16
Lu et al 2021	RCT	NCT03234972	141:70	≥1	DVd vs Vd	61	16
Mateos et al 2020	RCT	NCT02136134	251:247	≥1	DVd vs Vd	64	16

DRd = daratumumab + lenalidomide + dexamethasone, DPd = daratumumab + pomalidomide + dexamethasone, DVd = daratumumab + bortezomib + dexamethasone, Kd = carfilzomib + dexamethasone, KdD = carfilzomib + dexamethasone + daratumumab, Pd = pomalidomide + dexamethasone, RCT = randomized clinical trial, Rd = lenalidomide + dexamethasone, Vd = bortezomib + dexamethasone.

**Figure 2.**
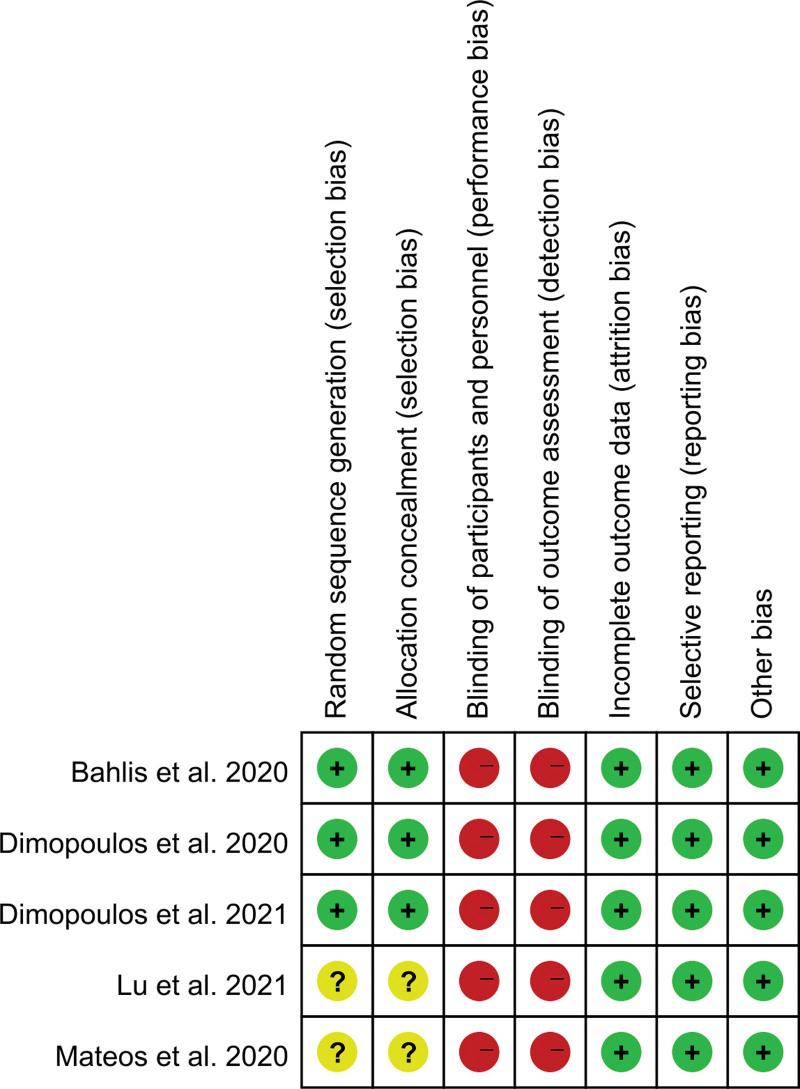
Summary of the risk of bias in the included studies.

The quality of the 5 included studies was evaluated using Review Manager 5.3 software (https://www.cochrane.org/). All 5 studies used random grouping, although 2 did not provide details about the random grouping method and 2 did not describe the allocation hiding method, which may have introduced selective bias. None of the 5 randomized controlled trials used blinding, which could have introduced implementation and measurement bias. Despite these limitations, the research results were considered accurate, reliable, and complete, and no other potential sources of bias were identified upon careful review of the literature. The results are presented in Figure 2.

### 3.3. Efficacy

#### 3.3.1. ORR

**Figure 3.**
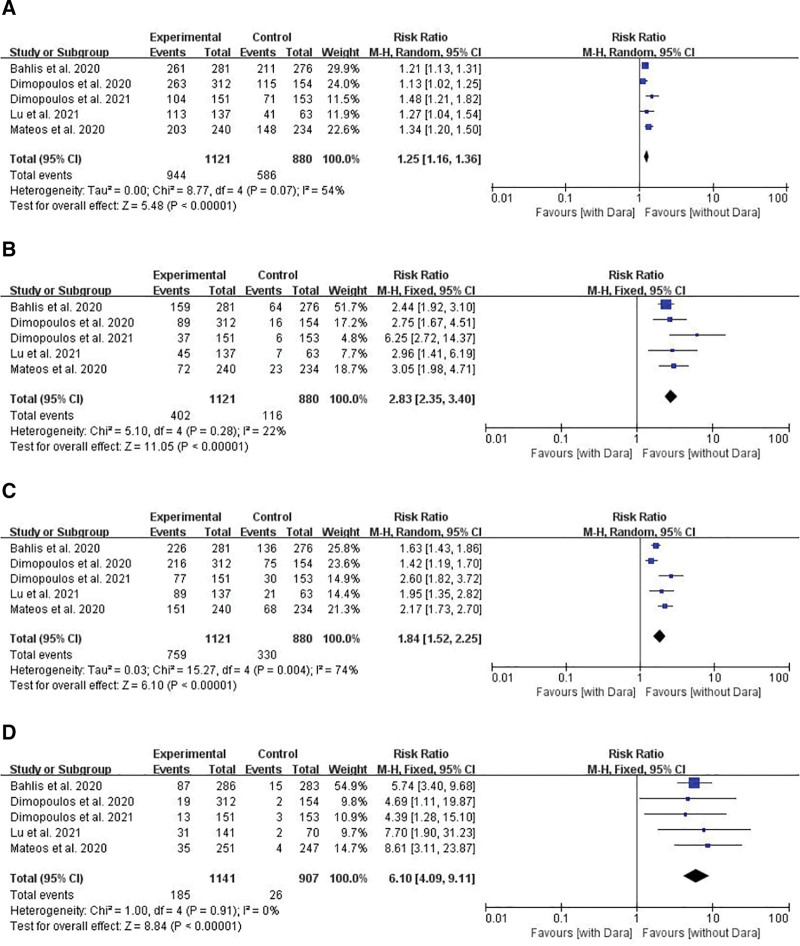
Meta-analysis of the efficacy of daratumumab groups compared with control groups (without daratumumab) in the treatment of multiple myeloma. (A) Comparison of the ORR between the two groups. (B) Comparison of the rate of complete response or better (≥CR) between the two groups. (C) Comparison of ≥ VGPR between the two groups. (D) Comparison of MRD between the two groups. MRD = minimal residual disease, ORR = overall response rate, VGPR = very good partial response.

The ORR was reported in all 5 studies.^[28–32]^ As there was moderate heterogeneity (*I*^2^ = 54%, *P* = .07), a random-effects model was used. The results showed that the ORR in the daratumumab group was significantly higher than that in the control group (RR = 1.25, 95% CI 1.16–1.36, *P* < .00001) (Fig. 3A)

#### 3.3.2. CR or better

All 5 studies^[28–32]^ reported the ≥ CR rate. A fixed-effects model was used as there was no clear evidence of heterogeneity (*I*^2^ = 22%, *P* = .28) among the trials. The results showed that the ≥ CR rate was significantly higher in the daratumumab group compared to the control group (RR = 2.83, 95% CI 2.35–3.40, *P* < .00001) (Fig. 3B).

#### 3.3.3. Very good partial response or better (≥VGPR)

The ≥ VGPR rate was reported in all 5 studies.^[28–32]^ However, there was significant heterogeneity (*I*^2^ = 74%, *P* = .004) among the reported VGPR rates, and therefore, the random-effects model was used for the meta-analysis. The results showed that the ≥ VGPR rate in the daratumumab group was significantly higher than that in the control group (RR = 1.84, 95% CI 1.52–2.25 *P* < .00001) (Fig. 3C).

#### 3.3.4. MRD

The MRD negativity rate was reported in all 5 studies.^[28–32]^ No significant heterogeneity was found with respect to the reported MRD negativity rate (*I*^2^ = 0%, *P* = .91), so the fixed-effects model was used. The results of meta-analysis showed that the MRD negativity rate in the daratumumab group was significantly higher than that in the control group (RR = 6.10, 95% CI 4.09–9.11, *P* < .00001) (Fig. 3D).

#### 3.3.5. PFS

**Figure 4.**
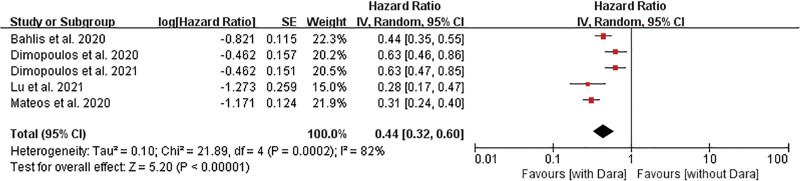
Forest plots of HRs for progression-free survival in the daratumumab and control groups. HR = hazard ratio.

PFS was reported in all 5 studies,^[28–32]^ but heterogeneity was found with respect to the reported PFS (*I*^2^ = 74%, *P* = .004), and thus, the random-effects model was selected. The meta-analysis results showed that the PFS in the daratumumab group was significantly higher than that in the control group (HR = 0.44, 95% CI 0.32–0.60, *P* < .00001) (Fig. 4).

### 3.4. Subgroup analyses

Subgroup analysis by the age of participant, ISS disease stage, type of measurable MM, baseline renal function level, cytogenetic profile, and number of prior lines of therapy was also conducted. The results of the further subgroup analysis showed that the PFS benefit was consistent between the treatment groups regarding the previous basic characteristics (Table 2 and Table 3).

**Table 2:** Analysis of basic characteristics for the effect of daratumumab for progression-free survival in subgroups.

Subgroup	R-arm	C-arm	Hazard ratio (95% CI)	*P* value	P for homogeneity
	Number of subjects				
Age					
≤65	598	446	0.46 [0.35, 0.60]	<.00001	0.07
>65	543	461	0.45 [0.31, 0.65]	<.00001	0.004
ISS disease stage					
I	375	339	0.36 [0.24, 0.53]	<.00001	0.03
II	282	259	0.41 [0.32, 0.51]	<.00001	0.33
III	172	155	0.54 [0.42, 0.70]	<.00001	0.32
Baseline renal function (CCr)					
>60 mL/min	596	539	0.40 [0.28, 0.57]	<.00001	0.003
≤60 mL/min	218	200	0.46 [0.35, 0.60]	<.00001	0.07
Cytogenetic profile					
High risk	208	158	0.53 [0.40, 0.72]	<.00001	0.16
Standard risk	594	482	0.38 [0.29, 0.51]	<.00001	0.08

C-arm = Control arm, CI = Confidence interval, R-arm = daratumumab-containing therapy arm.

**Table 3:** Analysis of previous therapy information for the effect of daratumumab for progression-free survival in subgroups.

Subgroup	R-arm	C-arm	Hazard Ratio (95% CI)	*P* value	P for homogeneity
	Number of subjects				
Number of prior lines of therapy					
I	461	333	0.36 [0.29, 0.45]	<.00001	0.002
II	200	179	0.43 [0.33, 0.57]	<.00001	0.75
III	100	78	0.55 [0.36, 0.84]	*P* = .006	0.88
> III	87	87	0.38 [0.25, 0.59]	<.00001	0.54

C-arm = Control arm, CI = Confidence interval, R-arm = daratumumab-containing therapy arm.

### 3.5. Safety

Data on major adverse events were extracted from the 5 RCTs and analyzed by a meta-analysis. Patients treated with daratumumab had a significantly higher rate of grade 3 to 4 neutropenia, lymphopenia, and thrombocytopenia, with neutropenia being the most common (30.5% vs 24.4%, *P* = .0006), followed by thrombocytopenia (29.4% vs 22.4%, *P* = .001). However, the results of a random-effects model meta-analysis showed that there was no statistical significance in the incidence of grade 3 to 4 anemia between the daratumumab group and the control group (RR = 0.96, 95% CI 0.79–1.16, *P* = .69) (Table 4). In terms of non-hematological toxicity, our analysis revealed higher incidences of pneumonia (RR = 1.43, 95% CI 1.16–1.76, *P* = .0008), upper respiratory tract infection (RR = 1.44, 95% CI 1.14–1.83, *P* = .003), and diarrhea (RR = 1.60, 95% CI 1.40–1.82, *P* < .00001). Due to insufficient data, only 3 clinical trials (NCT02076009, NCT02076009, NCT03180736) were used to analyze the association of daratumumab with second primary malignancies. The incidence of second primary malignancies in the daratumumab group was higher than that in the control group, but the difference was not statistically significant (RR = 1.24, 95% CI 0.80–1.93, *P* = .34) (Table 5).

**Table 4:** Summary of grade 3–4 hematological AEs related to daratumumab therapy.

Hematological AEs	Number of patients with available data	R-arm n/%	C-arm n/%	Exact RR (95% CI)	*P* value	P for homogeneity
Neutropenia	2003	340/30.5	217/24.4	1.51 [1.20, 1.92]	.0006	0.09
Lymphopenia	2003	140/12.6	54/6.1	1.79 [1.09, 2.92]	.02	0.04
Anemia	2003	199/17.9	165/18.6	0.96 [0.79, 1.16]	.69	0.52
Thrombocytopenia	2003	327/29.4	199/22.4	1.29 [1.11, 1.50]	.001	0.24

AEs = adverse events, C-arm = control arm, CI = confidence interval, R-arm = daratumumab-containing therapy arm, RR = risk ratio.

**Table 5:** Summary of non-hematological AEs related to daratumumab therapy.

Non-hematological AEs	Number of patients with available data	R-arm n/%	C-arm n/%	Exact RR (95% CI)	*P* value	P for homogeneity
Pneumonia	2003	208/18.7	120/13.5	1.43 [1.16, 1.76]	.0008	0.92
Upper respiratory tract infection	2003	383/34.7	204/22.9	1.44 [1.14, 1.83]	.003	0.04
Diarrhoea	2003	429/38.5	223/25.1	1.60 [1.40, 1.82]	<.00001	0.20
Constipation	1243	170/25.9	133/22.7	1.06 [0.70, 1.62]	.78	0.02
Hypertension	1140	155/22.7	53/11.6	2.47 [0.86, 7.10]	.09	0.001
Fatigue	1795	178/28.5	211/25.7	1.15 [0.98, 1.34]	.08	0.43
Second primary malignancies	1334	41/6.2	33/4.9	1.24 [0.80, 1.93]	.34	0.17

AEs = adverse events, C-arm = control arm, CI = confidence interval, R-arm = daratumumab-containing therapy arm, RR = risk ratio.

### 3.6. Sensitivity analysis

**Figure 5.**
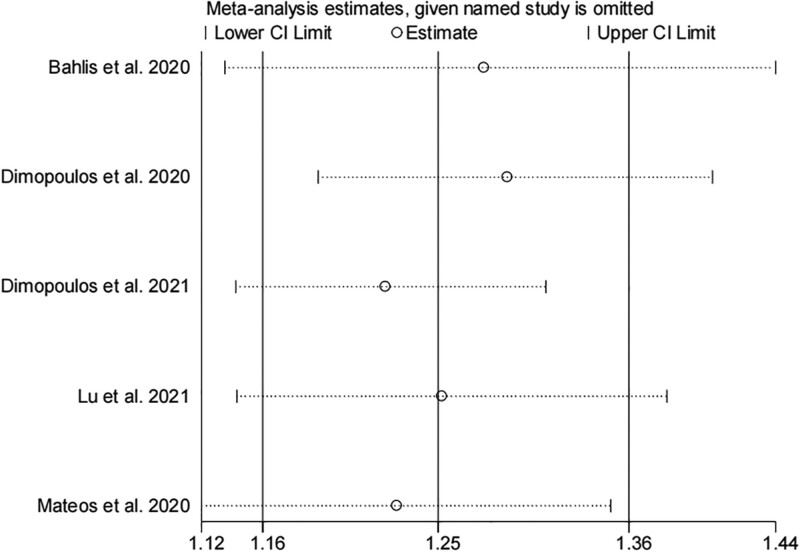
Sensitivity analysis for the comparison of ORR between the daratumumab and control groups, with each study omitted in turn. ORR = overall response rate.

Sensitivity analysis was performed using the “leave-1-out” approach to assess the stability of our results. As shown in Figure 5, each study was removed in turn from the pooled analysis, and the analysis indicated that the ORR was not significantly affected. The results of the analysis showed that the findings were overall stable.

## 4. Discussion

MM is a hematological malignancy characterized by clonal proliferation of plasma cells derived from the terminal differentiation of B cells.^[33,34]^ Despite advancements in treatment options, RRMM remain major challenges. CD38, a single-chain type II transmembrane glycoprotein, is significantly and consistently expressed on the surface of multiple myeloma cells.^[35]^ It serves various functions, including receptor-mediated adhesion, cell signaling, and regulation of cyclase and hydrolase activities.^[36]^ The high expression of CD38 on multiple myeloma cell membranes has made it an ideal therapeutic target for interventions in multiple myeloma.^[17]^ Consequently, the development of novel treatment drugs targeting CD38 has become a hot topic in RRMM management. Daratumumab, an IgGκ humanized monoclonal antibody, specifically binds to CD38.^[37]^ It induces cell apoptosis through Fc-mediated crosslinking and exerts immunomodulatory effects, such as antibody-dependent cell-mediated cytotoxicity, complement-dependent cytotoxicity, and antibody-dependent cellular phagocytosis.^[38,39]^ These mechanisms contribute to tumor cell lysis and the inhibition of CD38-expressing tumor cell growth. Daratumumab also acts on immune-suppressive cells, such as CD38 + regulatory T cells (Tregs), regulatory B cells (Bregs), and myeloid-derived suppressor cells, leading to the activation of cytotoxic CD8 + T cells and CD4 + helper T cells.^[19]^ Consequently, daratumumab indirectly contributes to the elimination of multiple myeloma cells by modulating the immune response. This study aimed to perform a meta-analysis of RCTs to evaluate the efficacy and safety of daratumumab in the treatment of RRMM. We conducted a search of major databases and clinical trial registration websites and identified 5 RCTs that met our inclusion criteria. A total of 2003 patients were randomly assigned to receive either daratumumab therapy or a control treatment. Our results indicated that patients treated with daratumumab had significantly improved rates of ORR, CR, VGPR, and MRD compared to the control group. Additionally, daratumumab treatment resulted in a significant prolongation of PFS compared to the control group.

However, when calculating the pooled HR for PFS, we found significant heterogeneity that we believe may have originated from several sources. First, in the PFS subgroup analysis, the heterogeneity may be attributed to differences in baseline creatinine clearance rate. Specifically, patients with a baseline creatinine clearance rate > 60 mL/minutes/1.73 m^2^ may have contributed to the heterogeneity, as this subgroup includes patients with both normal and abnormal renal function. Thus, renal function may be an important factor to consider when analyzing PFS data. Second, the 2 age subgroups included in the studies were heterogeneous, and we suggest that future clinical trials refine the age group of patients with RRMM. Moreover, heterogeneity in the ISS stage group originated from the phase I group, while heterogeneity in the previous treatment group was seen in patients who had received only 1 prior treatment. These subgroup analyses suggest that patients with less severe disease and fewer prior treatments may show greater efficacy when receiving daratumumab treatment. Despite these sources of heterogeneity, our results demonstrate that PFS was consistently longer in the daratumumab group than in the control group, regardless of patient subgroup.

Regarding the safety profile of daratumumab therapy, our pooled results indicated that cytopenias were the most frequent manifestation of hematological toxicities. Grade 3 or 4 neutropenia, thrombocytopenia, and lymphopenia were more common in the daratumumab therapy group than the control group, but there was no statistically significant difference in the incidence of grade 3 to 4 anemia between the 2 groups.

As for non-hematological toxicities, daratumumab therapy was associated with a higher rate of pneumonia, upper respiratory tract infection, and diarrhea. We suspect that the high incidence of infection in the daratumumab group may be related to neutropenia observed in hematologic adverse events. However, the incidences of fatigue, hypertension, second primary malignancies, and constipation were not significantly increased with daratumumab therapy.

The study has some limitations. Firstly, there is a lack of information regarding overall survival and whether daratumab provides a benefit in terms of patient survival. This uncertainty diminishes our understanding of daratumab’s impact on overall survival. Secondly, the sensitivity analysis conducted in the study was limited due to the small number of included studies. This limitation hinders the credibility of the results. To address these issues, future studies should focus on conducting large-scale, high-quality investigations to confirm and strengthen the findings.

## 5. Conclusion

In conclusion, our meta-analysis provides evidence that daratumumab-containing regimens are effective in improving the ORR, CR, and PFS in patients with RRMM. However, the increased risk of adverse events associated with daratumumab therapy should not be overlooked and requires careful consideration and management. Moreover, when choosing treatment regimens for RRMM, the head-to-head results of daratumumab compared to other drugs should be taken into account. Further studies are still needed to determine the optimal use of daratumumab in RRMM treatment and to assess its long-term safety and efficacy.

## Author contributions

**Conceptualization:** Zeng-Yi Huang, Qi-lian Liang.

**Data curation:** Zeng-Yi Huang, Xiao-Qin Jin, Ding-Yue Zhang, Han Han, Zhen-Wei Wang.

**Formal analysis:** Zeng-Yi Huang, Xiao-Qin Jin.

**Investigation:** Ding-Yue Zhang.

**Methodology:** Zeng-Yi Huang, Xiao-Qin Jin, Qi-lian Liang, Han Han, Zhen-Wei Wang.

**Project administration:** Zeng-Yi Huang, Qi-lian Liang.

**Resources:** Xiao-Qin Jin, Qi-lian Liang.

**Software:** Zeng-Yi Huang, Xiao-Qin Jin, Ding-Yue Zhang, Han Han, Zhen-Wei Wang.

**Supervision:** Qi-lian Liang.

**Validation:** Qi-lian Liang, Han Han.

**Visualization:** Qi-lian Liang.

**Writing – original draft:** Zeng-Yi Huang, Xiao-Qin Jin, Zhen-Wei Wang.

**Writing – review & editing:** Zeng-Yi Huang, Qi-lian Liang.
